# 2-Aminoethoxydiphenylborane sensitizes anti-tumor effect of bortezomib via suppression of calcium-mediated autophagy

**DOI:** 10.1038/s41419-018-0397-0

**Published:** 2018-03-02

**Authors:** Yuan Qing Qu, Flora Gordillo-Martinez, Betty Yuen Kwan Law, Yu Han, Anguo Wu, Wu Zeng, Wai Kei Lam, Charles Ho, Simon Wing Fai Mok, Hu Qiang He, Vincent Kam Wai Wong, Renxiao Wang

**Affiliations:** 1 State Key Laboratory of Quality Research in Chinese Medicine, Macau University of Science and Technology, Macau, People’s Republic of China; 2 Department of Pathology, University Hospital, Macau University of Science and Technology, Macau, People’s Republic of China; 3 State Key Laboratory of Bioorganic & Natural Products Chemistry, Shanghai Institute of Organic Chemistry, Chinese Academy of Sciences, Shanghai, People’s Republic of China

## Abstract

Non-small-cell lung cancer (NSCLC) accounts for most lung cancer cases. Therapeutic interventions integrating the use of different agents that focus on different targets are needed to overcome this set of diseases. The proteasome system has been demonstrated clinically as a potent therapeutic target for haematological cancers. However, promising preclinical data in solid tumors are yet to be confirmed in clinics. Herein, the combinational use of Bortezomib (BZM) and 2-aminoethoxydiphenylborane (2-APB) toward NSCLC cells was studied. We confirmed that BZM-triggered cytoprotective autophagy that may counteract with the cytotoxic effects of the drug per se. 2-APB was selected from screening of a commercial natural compounds library, which potentiated BZM-induced cytotoxicity. Such an enhancement effect was associated with 2-APB-mediated autophagy inhibition. In addition, we revealed that 2-APB suppressed calcium-induced autophagy in H1975 and A549 NSCLC cells. Interestingly, BZM [0.3 mg/kg/3 days] combined with 2-APB [2 mg/kg/day] significantly inhibited both primary (around 47% tumor growth) and metastatic Lewis lung carcinoma after a 20-day treatment. Our results suggested that BZM and 2-APB combination therapy can potentially be developed as a novel formulation for lung cancer treatment.

## Introduction

Lung cancer remains the leading cause of cancer deaths globally^1^, whereas non-small-cell lung cancer (NSCLC) is the most common type accounting for 85% of all lung cancers. Technological advances in genetics and signaling pathways analyses have further defined NSCLC as a group of distinct diseases with genetic and cellular heterogeneity^2^. Conventional treatment regimen includes surgery, radiotherapy, chemotherapy, or combinations thereof depending on disease stage. For example, platinum-based therapy is the mainstay chemotherapy and is usually given in combination with a tubulin binding agent^3^. Although most NSCLC patients are initially responsive to chemotherapy and the emergence of targeted therapy benefits the patients who harboured the specific genomic mutation, such as EGFR, intrinsic, or acquired resistance may eventually be developed over time^3,4^. Therefore, the management of NSCLC requires an integrated therapy with single or combined agents targeting multiple cellular compartments as the future of an effective lung cancer treatment^2,3^.

The ubiquitin–proteasome system (UPS) and autophagy are two major intracellular proteolytic systems^5^. The proteasome pathway plays a critical role in cellular homeostasis by degrading over 80% of the total proteins in cells, as well as misfolded or deleterious proteins maintaining the normal cellular physiology^5–7^. The proteasome system of malignant cells is usually overloaded by the accumulation of defective proteins^6,7^. Consequently, a novel pathway to cancer therapy associated with the use of proteasome inhibitors has been proposed. Bortezomib (BZM), also called as VELCADE® and formerly known as PS-34, was the first FDA-approved proteasome inhibitor for the treatment of relapsed/refractory multiple myeloma disease owing to its impressive clinical activity. The efficacy of the proteasome inhibitor has been evaluated in various cancer models with different combinations that highlighted several properties of this type of agents that render them suitable for anticancer therapy^7,8^. However, relapses are frequent and acquired resistance to treatment ultimately emerges, especially in models of solid tumors, which implies the importance of primary resistance^7–9^. The poor in vivo efficacy as observed in BZM-treated solid tumors may be mediated by the activation of autophagy pathway that functions as an alternative mechanism of protein degradation assisting cancer cell survival via the relief in proteotoxic stress^10–16^. BZM causes the accumulation of misfolded and ubiquitinated proteins that eventually lead to endoplasmic reticulum (ER) stress and increase intracellular calcium (Ca^2+^) release that activates autophagy^16–22^. Autophagy is a highly conservative system in eukaryotic cells regulated by the autophagic genes (*Atg*) involving the engulfment of unwanted proteins or damaged organelles into the autophagosomes that are eventually delivered to the lysosome for degradation^23,24^. Thus far, the only clinically approved autophagy inhibitor is an anti-malarial chloroquine and its derivatives, such as hydroxychloroquine (HCQ). HCQ inhibits autophagy by hampering lysosomal acidification that prevents autophagosomal degradation. Preclinical trials have shown that HCQ in combination therapy leads to enhancement of antineoplastic effects in different cancer models, including BZM for myeloma. However, HCQ displays reduced potency in vivo, thus creating a demand for the production of more potent autophagy inhibitors^25^.

In this study, we aimed at identifying novel autophagy inhibitors through Ca^2+^ modulation. We confirmed that BZM alone induced cytoprotective autophagy therefore, it reduced the therapeutic efficacy per se. However, we have identified the compound 2-Aminoethyl diphenylborinate (2-APB) from the screening of natural compounds library, which was able to sensitize BZM treatment in NSCLC cell lines. In addition, we further evaluated the corresponding cytotoxicity, autophagy inhibitory effect, and the underpinning mechanism induced by such BZM-2-APB simultaneous treatment. Finally, the anti-tumor effect of BZM in combination with 2-APB was confirmed in a murine lung cancer model.

## Results

### BZM induces cytoprotective autophagy

**Fig. 1:**
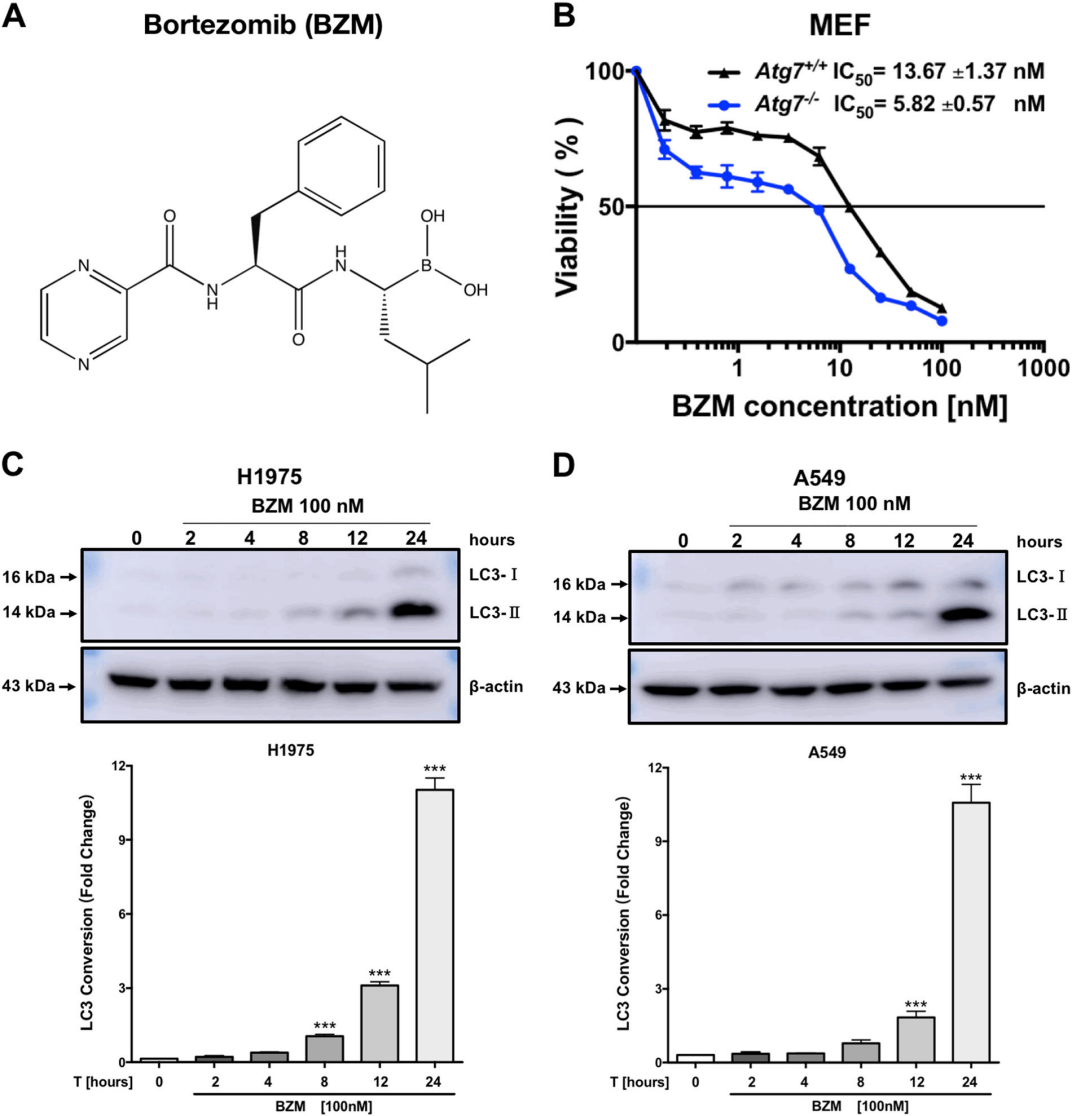
**BZM induces cytoprotective autophagy.** **a** Chemical structure of Bortezomib (BZM). **b** BZM illustrated higher cytotoxicity in autophagy-deficient *Atg7* MEF model cells. The percentage of viability was performed by cell cytotoxicity assay. Data are mean (*n* = 3) ± S.D. **c**, **d** BZM induced autophagy in H1975 and A549 lung cancer cell lines, respectively. Cells were treated with BZM [100 nM] for 0, 2, 4, 8, 12, and 24 h. Representative immunoblots and the LC3-II conversion quantification (*n* = 3). Data are mean ± S.D. ***P ≤ 0.001, one-way ANOVA analysis

Targeting the proteasome pathway has shown a promising new cancer strategy^7,8^. However, the decreased efficacy in clinics treating solid tumors could be mediated by autophagy activation as a resistant mechanism. BZM is a dipeptide boronic acid derivative (Fig. 1a) that binds in reversible fashion to the β5 subunit, inhibiting the 20S catalytic core particle^6^. To confirm whether BZM treatment triggers protective autophagy, cell viability was analyzed in *Atg7*-deficient cell model. As shown in Fig. 1b, *Atg7-*deficient MEF cells showed significantly higher BZM cell toxicity (2.35-fold) compared with the wild-type *Atg7* MEF cells. Thus, BZM displayed more cytotoxic potency in the autophagy-deficient cells when compared with the wild-type counterpart, suggesting that proteasome inhibition therapy triggered cytoprotective autophagy. To further demonstrate whether BZM induces autophagy in NSCLC cells, the specific autophagy biomarker microtubule-associated protein 1A/1B-light chain 3 (LC3) was tested in H1975 and A549 cell lines by immunoblot analysis. BZM treatment at 100 nM was selected according to previous studies^26^ in a time-dependent manner. The immunoblotting images showed an increasing LC3-phosphatidylethanolamine conjugate (LC3-II) expression in both NSCLC cell lines reaching the maximum level after 24 h, thus demonstrating that BZM-induced autophagy (Fig. 1c, d). These findings confirmed previous observations by others that BZM activated a cytoprotective autophagy.

### 2-APB sensitizes BZM-mediated cytotoxicity in a panel of cancer cells

**Fig. 2:**
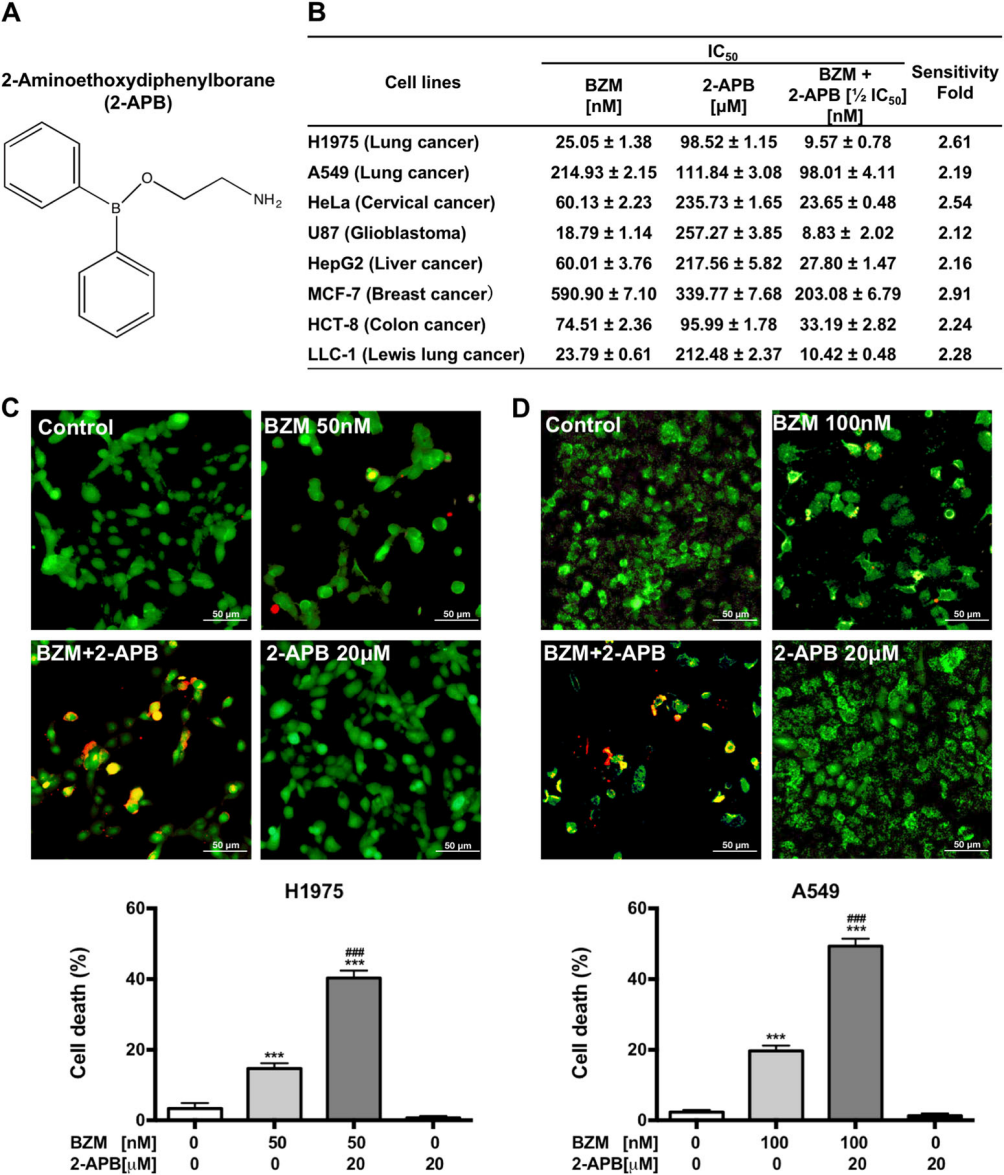
**2-APB sensitizes BZM cytotoxicity in a panel of cancer cells.** **a** Chemical structure of 2-Aminoethoxydiphenylborane (2-APB). **b** 2-APB potentiated the cytotoxic effect of BZM in a panel of cancer cells as indicated. Data are IC_50_ mean (*n* = 3) ± S.D. **c**, **d** 2-APB enhanced BZM-mediated cytotoxicity in H1975 and A549 NSCLC cell lines, respectively. Cells were co-treated with BZM and 2-APB for 48 h following 2-APB pre-treatment for 2 h as indicated. Data represent the percentages of cell death analyzed by LIVE/DEAD cells analysis (mean ± S.D., *n* = 3). *P < 0.05, ***P ≤ 0.001 compared to control (untreated). ^#^*P* < 0.05, ^##^*P* ≤ 0.01 compared to single BZM treatment, one-way ANOVA analysis

To increase the anti-cancer effect of BZM, 800 compounds library were screened to identify those non-toxic compounds capable of improving the efficacy of BZM in H1975 cell line (Supplementary Fig. 1). A range of dosage (1.56–50 µM) was set to select those compounds with a better therapeutic index. Firstly, the compounds with an IC_50_ value <10 µM were excluded to avoid further side effects. Then, only those compounds able to potentiate the BZM anti-tumor effect (sensitivity fold) by more than twice were selected. Three compounds with CAS. 524-95-8 (2-APB), CAS. 846-46-8 (5alpha-androstan-3,17-dione), and CAS. 57-06-7 (allyl isothiocyanate) showed 2.51, 2.17, and 2.10 sensitivity fold, respectively. 2-APB, a diphenylborate derivative (Fig. 2a) is a cellular Ca^2+^ modulator^27^, was chosen for further studies owing to its potent BZM inhibitory effect and its feasibility to be an autophagy inhibitor through the regulation of Ca^2+^. Furthermore, the drug combination was tested and calculations of combination index (CI) values using CompuSyn software^28^ indicated significant synergistic growth inhibition (CI < 1) effects between 2-APB and BTZ (Supplementary Fig. 2).

To evaluate whether 2-APB combined with BZM was capable of improving BZM treatment efficacy in different cancer models, a panel of cancer cells from different origins was used including A549 (NSCLC), HeLa (cervical cancer), U87 (glioblastoma), HepG2 (liver), MCF7 (breast), HCT8 (colon) human cell lines, and LLC-1 (Lewis lung cancer) mouse cell line. As illustrated in Fig. 2b, BZM displayed an extensive cytotoxicity in the nanomolar range in all cancer cells tested, whereas 2-APB showed a relative low cytotoxicity in the micromolar range. In the combination, 2-APB enhanced BZM sensitivity by more than twice, increasing the cytotoxic effect in all cell lines tested. To further validate the anti-tumor effect of the combinatorial therapy and the therapeutic index of 2-APB in NSCLC model, cell death studies were performed by LIVE/DEAD analysis in H1975 and A549 cell lines reducing the amount of 2-APB to 20 µM. As shown in Fig. 2c, d, the cytotoxicity of BZM, as single agent or combined with 2-APB treatment, was increased in both H1975 and A549 cells compared to control. In addition, the combination therapy increased the BZM effect by over two fold compared to BZM treatment alone, even at low concentration of 2-APB, indicating its potential therapeutic index and effectiveness in the combination treatment.

### 2-APB inhibits BZM-induced autophagy in H1975 and A549 NSCLC cells

**Fig. 3:**
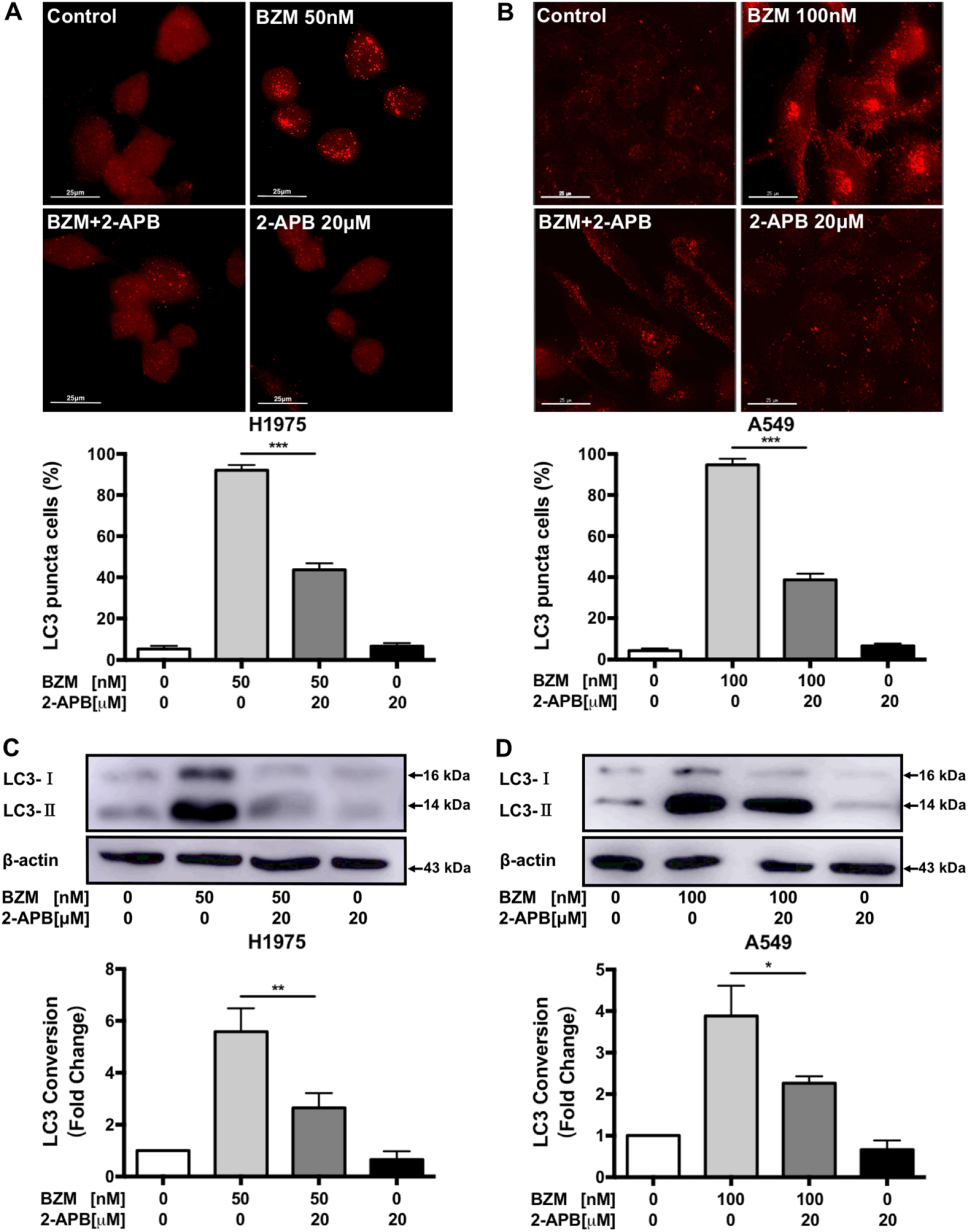
**2-APB inhibits BZM-induced autophagy in H1975 and A549 NSCLC cells.** Cells were co-treated with BZM and 2-APB for 24 h following 2-APB pre-treatment for 2 h as indicated. **a**, **b** 2-APB decreased LC3-II puncta formation induced by BZM in H1975 and A549 cell lines, respectively. Representative micrographs of cells with endogenous LC3-II puncta and the percentages of cells with increased LC3-II puncta formation are displayed in the upper and bottom panels, respectively. Scale bar = 25 µm, ×60. **c**, **d** 2-APB reduced LC3-II conversion induced by BZM in H1975 and A549 cell lines respectively. Representative immunoblots and the LC3 conversion quantification are shown in the upper and bottom panels, respectively. Data are mean value ± S.D. (*n* = 3). *P < 0.05, **P ≤ 0.01, ***P ≤ 0.001, one-way ANOVA analysis

To investigate whether 2-APB enhanced BZM treatment efficacy was correlated with autophagic activity suppression, the percentage of endogenous autophagic LC3-II puncta cells quantification was determined by immunofluorescence in H1975 and A549 NSCLC cells. As illustrated in Fig. 3a, b, BZM treatment induced autophagy as indicated due to increased percentages of red endogenous LC3-II puncta formation (red TRITC signal), whereas the control cells showed only slight or no red puncta formation. And 2-APB was found to significantly reduce the percentage of autophagic cells by half in the combination with BZM. Alternatively, the 2-APB-mediated autophagy inhibition was further supported by immunoblot analysis. Accordingly, 2-APB distinctly decreased the LC3-II conversion in combined BZM treatment (Fig. 3c, d) as the endogenous LC3-II puncta detection. These results suggested that 2-APB inhibited BZM-induced autophagy in H1975 and A549 NSCLC cells.

### 2-APB inhibits basal autophagy in HeLa, H1975, and A549 cancer cells

**Fig. 4:**
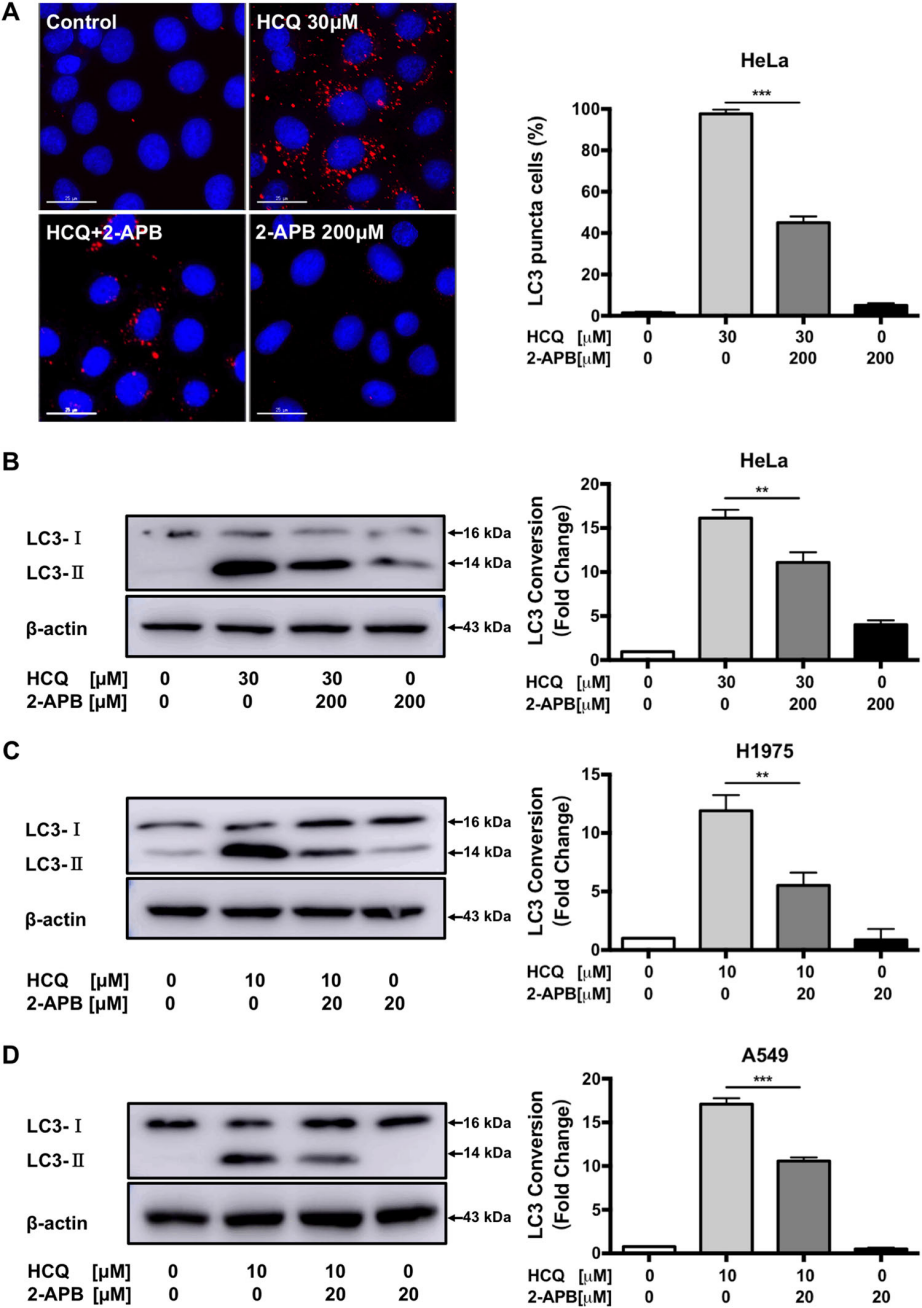
**2-APB inhibits basal autophagy in HeLa, H1975, and A549 cancer cells.** After pre-treatment of 2-APB for 2 h, the cells were then co-treated with HCQ and 2-APB for another 24 h. **a** 2-APB reduced LC3-II puncta accumulated by HCQ in HeLa cell line. Representative micrographs of cells with endogenous LC3-II puncta and the percentage of cells with increased endogenous LC3-II puncta formation are shown. DAPI was used for counterstaining the nucleus. Scale bar = 25 µm, ×60. **b** 2-APB decreased LC3-II conversion accumulated by HCQ in HeLa cells. Representative immunoblots and the LC3 conversion quantification are displayed. **c**, **d** 2-APB reduced LC3-II conversion accumulated by HCQ in H1975 and A549 cell lines, respectively. Representative immunoblot and LC3 conversion quantification are shown; *n* = 3; **P ≤ 0.01, ***P ≤ 0.001, one-way ANOVA analysis

Emerging lines of evidence indicate that autophagy plays a prosurvival role in cancer to overcome microenvironmental stress and maintain their rapid proliferation. Many tumor cell types demonstrate elevated basal autophagy even under nutrient-supplied conditions^29^. To investigate how 2-APB inhibits autophagy, the basal autophagy inhibition was further assessed by applying HCQ to prevent autophagosomal degradation. Based on our previous studies, HeLa cancer cells with flattened cell morphology and distinct cellular compartment represent an appropriate model for the study of autophagy process^30^. As shown in Fig. 4a, the well-known lysosomal blocker HCQ revealed the basal autophagy activity in HeLa cancer cells^31^. The addition of 2-APB markedly suppressed the HCQ-mediated accumulation of autophagosome, indicating that the basal autophagy activity was significantly inhibited. Consistently, the LC3-II conversion was reduced remarkably in the combination when compared to HCQ alone in HeLa cells (Fig. 4b). In relation to these results, 2-APB combined with HCQ decreased LC3-II conversion and prevented autophagosome accumulation in H1975 and A549 NSCLC cells (Fig. 4c,d). Collectively, these results suggest that 2-APB inhibited autophagy at early stages of the autophagy process.

### 2-APB suppresses BZM-induced intracellular Ca^2+^ mobilization in NSCLC cells

**Fig. 5:**
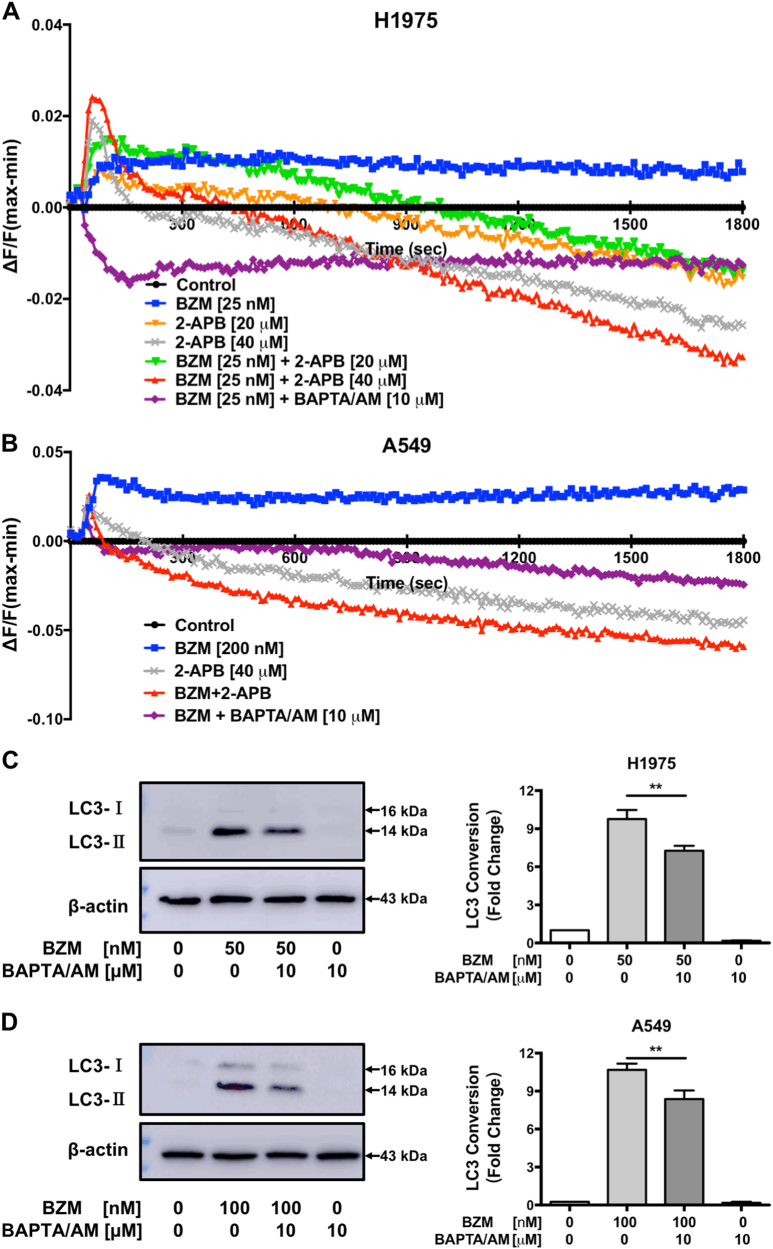
**2-APB inhibits BZM-mediated autophagy through intracellular Ca**^**2+**^**mobilization suppression in NSCLC cells.** **a**, **b** Intracellular Ca^2+^ dynamic changes were performed in H1975 and A549 cell lines, respectively. Cells were loaded with FLIPR Calcium 6 dye and treated as indicated. Real-time Ca^2+^ kinetic was monitored with FLIPR Tetra instrument. Data represent mean values of four independent samples. **c**, **d** BAPTA/AM reduced LC3-II conversion induced by BZM in H1975 and A549 cell lines respectively. Cells were co-treated with BZM and BAPTA/AM for 24 h following BAPTA/AM pre-treatment for 2 h as indicated. Representative immunoblot and LC3 conversion quantification are shown; *n* = 3; **P ≤ 0.01, one-way ANOVA analysis

Previous studies have shown that 2-APB is a reliable blocker of store-operated Ca^2+^ (SOC) channel^27^. The activation of SOC channel is crucial in mediating cytosolic Ca^2+^ signals and homeostatic control of both cytosolic and ER luminal Ca^2+^ levels. To further elucidate whether the mechanism of autophagy inhibition by 2-APB is related to cytosolic Ca^2+^ mobilization, real-time intracellular Ca^2+^ dynamic change was monitored in H1975 and A549 NSCLC cells. As illustrated in Fig. 5a, BZM treatment exhibited an increasing and persistent cytosolic Ca^2+^ flux indicating ER stress. However, 2-APB alone and in combination with BZM treatment, the intracellular Ca^2+^ concentration decreased following a transitory cytosolic Ca^2+^ flux in a dose-dependent manner suggesting that 2-APB suppressed BZM intracellular signaling in H1975 cell line. As expected, the positive control treatment with BAPTA/AM (Ca^2+^ chelator) combined with BZM suppressed cytosolic Ca^2+^ mobilization. Concomitantly, A549 showed the same Ca^2+^ dynamic changes tendency (Fig. 5b). To investigate whether the 2-APB autophagy inhibition mechanism was mediated by intracellular Ca^2+^ suppression, the LC3-II conversion induced by BZM was analyzed in the presence of BAPTA/AM. In line with the Ca^2+^ real-time detection, BAPTA/AM decreased the BZM-induced autophagy significantly as indicated by the LC3-II conversion (Fig. 5c, d). Therefore, these observations supported that the 2-APB autophagy inhibition mechanism was mediated by the suppression of intracellular Ca^2+^ mobilization in H1975 and A549 cells.

### BZM and 2-APB concomitant treatment potentiates the anti-tumor effect in LLC-1 bearing C57BL/6 mouse model

**Fig. 6:**
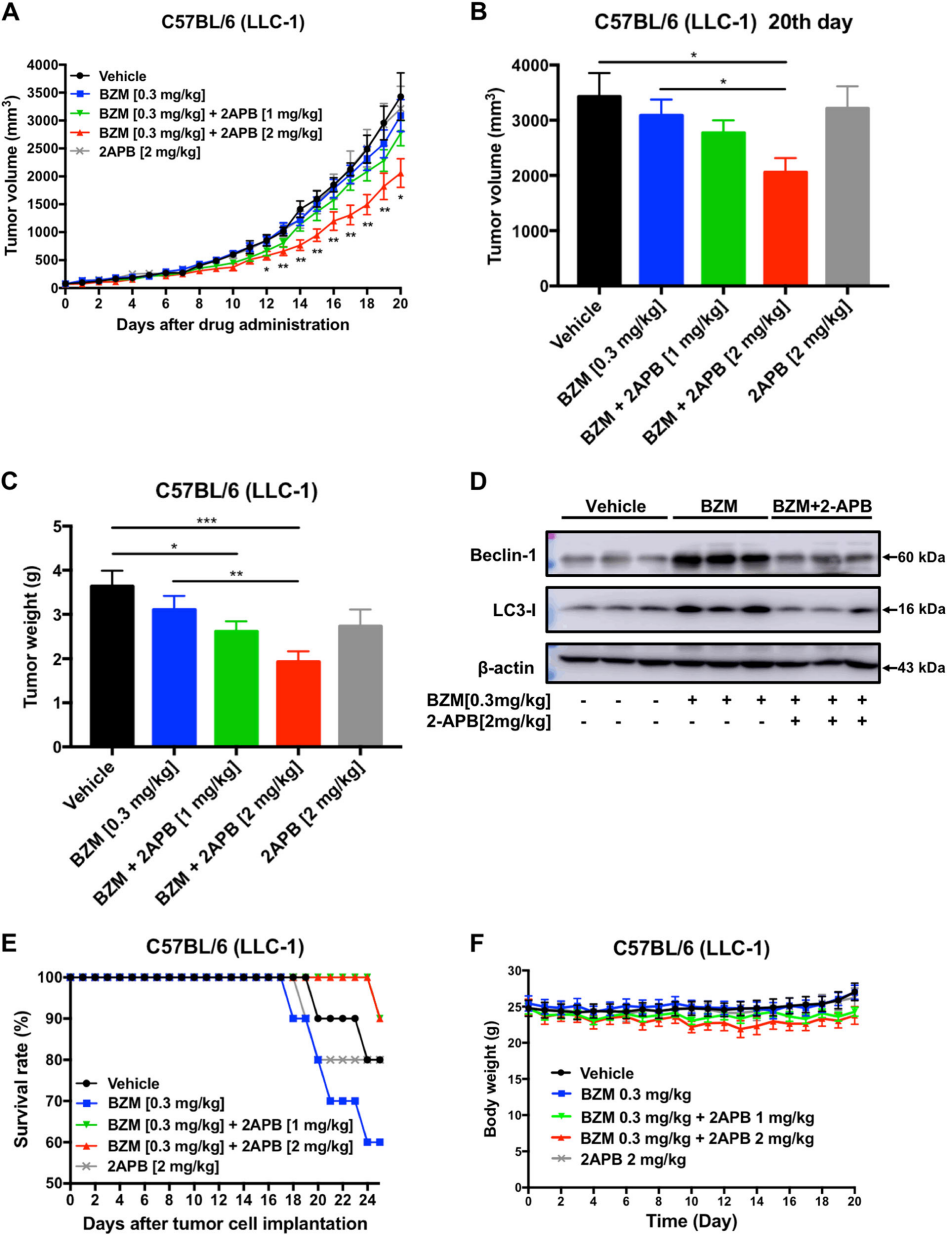
**BZM and 2-APB concomitant treatment suppresses tumor growth in LLC-1 bearing C57BL/6 mouse model.** After tumor establishment, mice were treated with BZM [0.3 mg/kg/3 days] and/or 2-APB [1 or 2 mg/kg/day] combinations via I.P. injections as indicated for 20 days (*n* = 10, both male and female). **a**–**c** Simultaneous treatment of 2-APB [2 mg/kg/day] and BZM enhanced tumor growth inhibition. Mice tumor volume monitorization, statistics of tumor volume after drug administration, and mice tumor weight are displayed respectively. **d** 2-APB increased BZM treatment efficacy through autophagy inhibition. Tumor tissues lysates were analyzed by western blot for LC3, Beclin-1, and β-actin (as loading control). **e** BZM in combination with 2-APB improved the survival rate of C57BL/6-LLC-1 bearing mice compared with BZM group. The lifespan data was collected over the course of the experiment. **f** Mice body weight monitorization is shown. All data represent mean ± SEM. *P < 0.05, **P ≤ 0.01, ***P ≤ 0.001, Student’s *t*-test

**Fig. 7:**
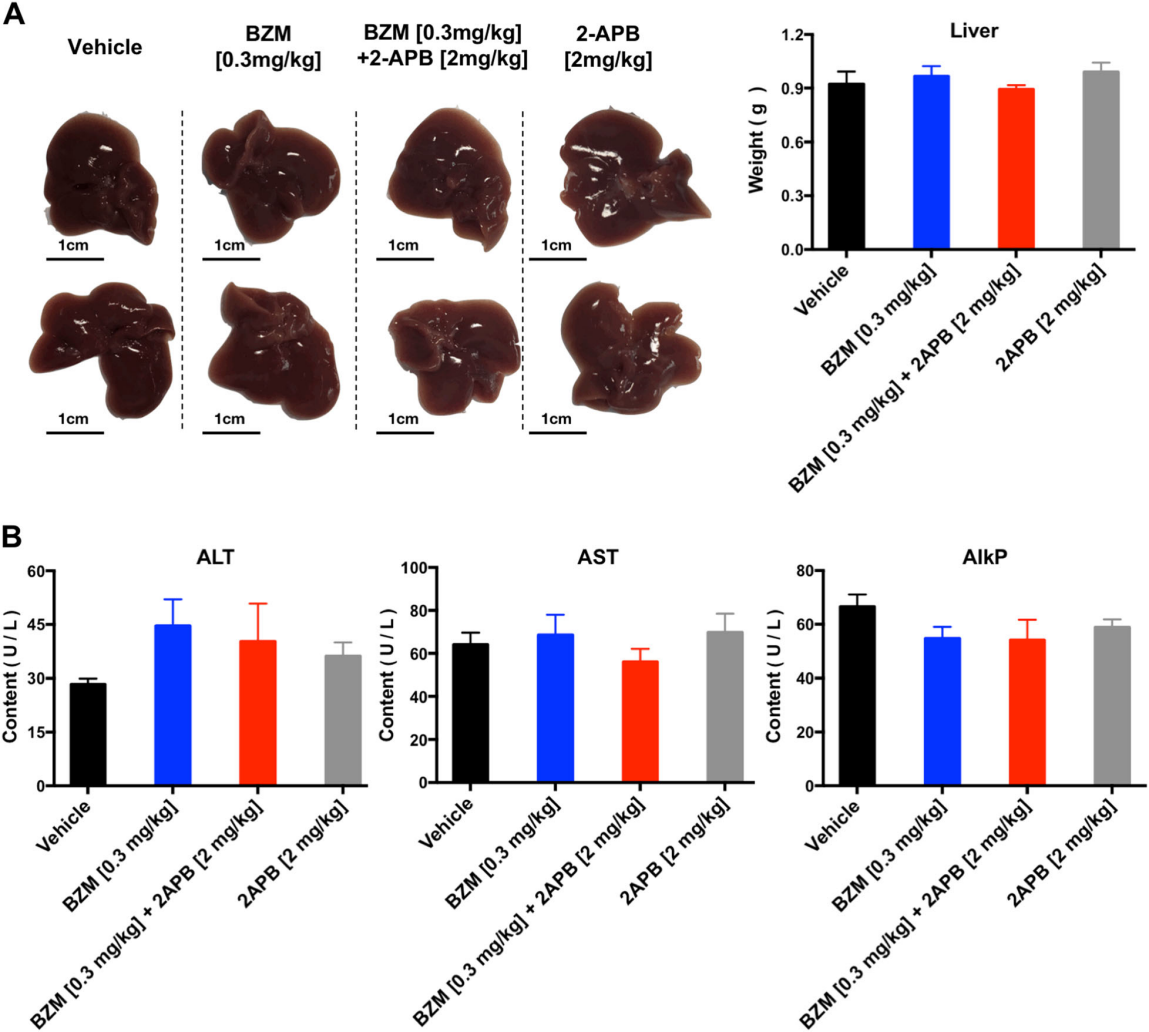
**Simultaneous treatment of 2-APB and BZM do not cause significant liver toxicity.** Twenty-four mice were randomly divided into four groups as indicated and were treated following the treatment regimen in Supplementary figure 2. **a** Representative images of liver and weights. **b** Serum alanine transaminase (ALT), aspartate transaminase (AST), and alkaline phosphatase (AlkP) levels of drug-treated mice. All data are mean ± SEM from 6 mice (3 males and 3 females) per treatment group. *P < 0.05, **P ≤ 0.01, ***P ≤ 0.001, one-way ANOVA analysis

**Fig. 8:**
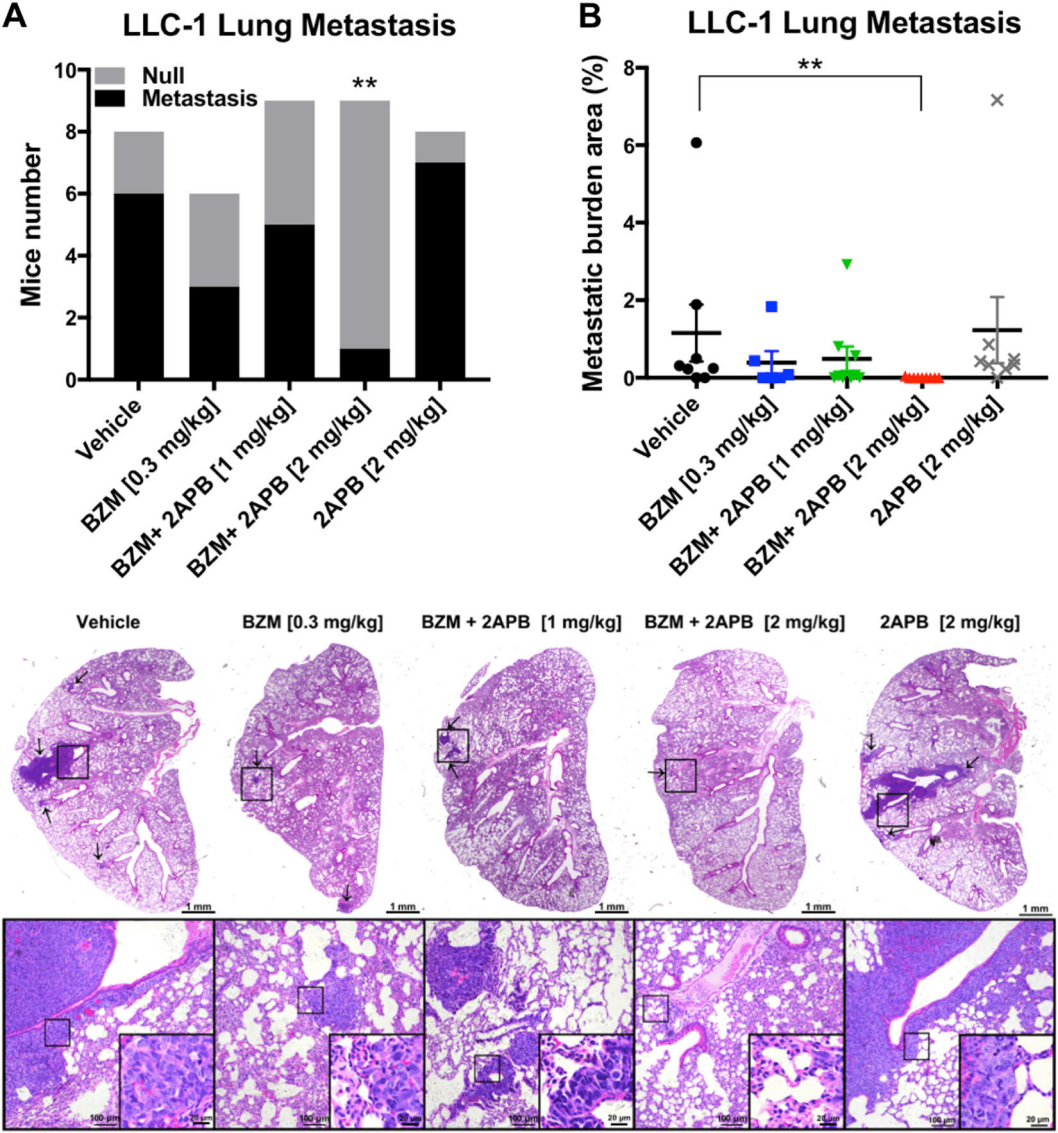
**Simultaneous treatment of 2-APB and BZM decreases the lung metastasis burden in LLC-1 bearing C57BL/6 mouse model.** **a** BZM and 2-APB [2 mg/kg/day] co-treatment decreased the lung metastasis frequency. The bar chart represents the number of mice that survived in each group. Number of mice with lung metastatic lesions (black area) and mice without metastatic lesion (gray area) are shown. *P < 0.05, **P ≤ 0.01, ***P ≤ 0.001, Chi-square test. **b**, **c** BZM and 2-APB [2 mg/kg/day] combination reduced the metastatic burden area. The percentage of metastatic burden area is displayed in **b**. Each dot represents one mouse. Data represent mean ± SEM. *P < 0.05, **P ≤ 0.01, ***P ≤ 0.001, Mann–Whitney *t*-test. Representative H&E stained lung sections images with metastatic lesions are shown in **c**. Areas of metastatic lesions are shown with arrows, and infiltrating metastatic cells are detailed at higher magnification. Scale bar = 1 mm (×2.5), scale bar = 100 µm (×10), and scale bar = 20 µm (×40)

To further confirm the potential anti-tumor effect of BZM and 2-APB combination in lung cancer, a mouse lung cancer model was evaluated. C57BL/6 mice were selected for establishing the LLC-1 animal model which has been suggested as a suitable preclinical experimental platform used to predict clinical benefit of lung cancer therapy by evaluating the metastasis profile, reliable toxicity responses, and the efficacy of chemotherapeutic agents^32^. We decided to administrate 0.3 mg/kg dosage of BZM based on the Marten et al. study^9^. And accordingly, with prior rat assessment, 2-APB minimum effective dose [1 mg/kg] was converted to mouse [2 mg/kg] and was administrated every day^9,33^. After tumor establishment, BZM and 2-APB were administrated by intraperitoneal injection following the regimen shown in Supplementary Fig. 2. As shown in Fig. 6a, tumor growth patterns displayed that the combination of BZM with 2-APB at 2 mg/kg efficiently inhibited tumor growth from day 12 to day 20 after drug administration. The tumor volumes were significantly reduced in the BZM group combined with a high dosage of 2-APB when compared to the control and BZM groups after treatment (Fig. 6b). Especially, tumor weights remarkably decreased in BZM-2-APB combination therapy in a dose-dependent manner reaching 28.19 and 46.91 percentage of tumor growth inhibition in 2-APB 1 and 2 mg/kg, respectively (Fig. 6c). However, as single-agent 2-APB did not inhibit tumor growth consistently with the cytotoxicity studies in vitro, neither did BZM treatment, unexpectedly. The protein lysate from tumor tissues indicated that both LC3 and Beclin-1 expressions were downregulated in combined BZM-2-APB group compared to BZM treatment alone, these findings further supported that the mechanism of sensitizing the anti-tumor effect of BZM was mediated by 2-APB autophagy suppression (Fig. 6d). Notably, the survival rate of the tumor-bearing mice was improved in the combined treatment when compared to the single BZM treatment group independently of 2-APB dosage (Fig. 6e). Although there was a clear drop in the survival rate with the BZM-treated group compared to the vehicle alone, during the treatment period, no significant change in body weight (Fig. 6f), liver weight (Fig. 7a), or the levels of liver enzymes in serum (Fig. 7b) were observed, indicating that the applied doses of BZM and 2-APB did not cause evident toxicity. Interestingly, the combination therapy had antitumor activity against both primary and metastatic disease. As shown in Fig. 8a, the number of mice with lung metastasis decreased in the combined therapy with a high dosage of 2-APB (1 metastatic lesion within 9 mice) compared to vehicle (6 metastatic lesions within 8 mice) or 2-APB groups (7 metastatic lesions within 8 mice). Whereas, the metastatic lesions frequency was slightly reduced in the combination with low dosage of 2-APB (5 metastatic lesions within 9 mice) and BZM groups (3 metastatic lesions within 6 mice). In addition, the size of the resulting lung metastatic lesions was quantified recapitulating the same tendency that was observed in metastasis frequency (Fig. 8b, c). The average of metastatic burden area per group was 0.005, 0.489, 0.393, 1.229, and 1.155% for the combination therapy with high dosage of 2-APB [2 mg/kg], combination with low dosage [1 mg/kg], BZM [0.3 mg/kg], 2-APB [2 mg/kg] and vehicle, respectively. These results demonstrated the efficacy of BZM-2-APB combination therapy in vivo by inhibiting tumor growth, enhancing BZM survival rate, and dramatically retarding lung tumor metastatic progression by autophagy suppression.

## Discussion

In the present study, we confirmed that BZM therapy can trigger autophagy which therefore may protect cancer cells from BZM-induced cell death. In fact, studies have shown that the reversible inhibition of 20S proteasome by BZM causes the accumulation of misfolded and ubiquitinated proteins that eventually lead to ER stress triggering the unfold protein response (UPR), formation of aggresomes, as well as autophagy activation^20–22^. Although the detailed intracellular network that orchestrates such protein quality control systems remains elusive, increasing evidence has illustrated that combination of proteasome and autophagy inhibitors can improve the therapy efficacy^34^. Herein, a novel autophagy inhibitor 2-APB was identified from a panel of 800 natural compounds, which can enhance the cytotoxicity induced by BZM toward cancers.

Mechanistically, we proved that 2-APB suppressed intracellular Ca^2+^ mobilization induced by BZM in NSCLC cells indicating that the autophagy inhibition could be mediated by Ca^2+^ repression. In fact, previous works revealed that BZM induces ER-stress and increases intracellular Ca^2+^ release which activates autophagy through the CAMKK2 (calcium/calmodulin-dependent protein kinase kinase 2) pathway^16–19^. In our experimental models, 2-APB could inhibit BZM-induced autophagy through the blockage of ER Ca^2+^ influx, since the compound is a powerful functional modifier of membrane-bound SOC^27,35,36^. The activation of SOC channels mediates store-operated Ca^2+^ entry (SOCE) that is crucial to replenish cytosolic Ca^2+^ stores, therefore, the homeostatic control of both cytosolic and ER luminal Ca^2+^ levels. The stromal interaction molecule 1 (SITM1) in the ER is a cellular sensor of luminal level of Ca^2+^. Upon ER luminal Ca^2+^ depletion, STIM1 will be activated, oligomerized, and tethered with SOC that leads eventually to the manipulation of intracellular Ca^2+^ regulation^19,35,37^. Therefore, 2-APB may interact with the SOC-STIM1 signaling and regulate the autophagic response in a Ca^2+^-dependent manner.

Furthermore, we also illustrated that 2-APB inhibited the basal autophagy level. Autophagy functions as a double-edged sword in cancer, maintaining proliferation and survival since it prevents environmental stress (such as hypoxia, nutrient deprivation, and therapies) on the one hand and persistent activation causes autophagic programmed cell death on the other^38–43^. Generally, there are more small-molecule inducers of autophagy than inhibitors at various stages of development^30,31,44^. Some of the new autophagy inhibitors target different points in the autophagic pathway, for example ROC325 and Lys05 target the lysosome. While 3-Methyladenine, SBI-0206965, Spautin-1, and NSC185058 target the autophagosome formation^25,31^. We proved that 2-APB targets the autophagic pathway at early stages by reducing the autophagosome formation through the modulation of cellular Ca^2+^, thus constituting a new mechanism for autophagy inhibition compared to other autophagy inhibitors. Numerous studies have shown that cytosolic Ca^2+^ signals can control the autophagy machinery at various stages of autophagic flux^45,46^. Although the 2-APB-induced autophagy inhibition should be evaluated under different environmental stress and types of cancers, the control of autophagy modulating Ca^2+^ channel could constitute a potential target for cancer therapy.

Finally, we demonstrated the superiority of the drugs combination antitumor activity against both primary and metastatic compared to the single agents in Lewis lung carcinoma model. The BZM illustrated minimal antitumor effects when used alone which is consistent with other preclinical data acquired from treatment tests of different solid cancers^9,47–50^. Our animal data for BZM as monotherapy are in contrast to the tumor-growth delay assay from Teicher et al.^51^ and Lewis lung carcinoma model. They were administrated at the beginning on the same day of tumor implantation, after 4 days (angiogenesis starts), or after 7 days (well established) decreasing the anti-tumor activity depends on the stage of tumor development after 12 days when tumors reach 500 mm^3^. Therefore, probably this difference is due to the higher tumor burden in our animal model. Despite proteasome inhibitors having over a 20-year history in cancer therapy they are only available for hematological therapy in clinics^52^. Some combinations with conventional chemotherapies have been proposed^51^, however, the resulting additive toxicity from these combinations therapy can cause even more side effects in patients. We revealed that 2-APB did not exert any therapeutic effects when it was applied as a single agent, implying a low cytotoxicity of the compound. The most common side effects of BZM treatment are peripheral neuropathy and thrombocytopenia^53^, and it has been shown that it exerts species-specific toxicity in mice^54^, which may account for the poor survival in our BZM-treated animal group. In contrast, the use of BZM at 0.3 mg/kg and 2-APB combinatorial therapy improved the survival outcome. All these data point to the notion that, via autophagy inhibition, 2-APB could be an ideal enhancer for supporting cancer therapy using BZM without eliciting severe side effects. In addition, our preliminary data using glioblastoma, cervical, liver, breast, and colon cancer cell lines indicate the possibility of offering a broader impact in cancer therapy.

Taken together, combined proteasome (BZM) and autophagy inhibitors through Ca^2+^ channel (2-APB) therapy leads to enhancement of tumor shrinkage and metastasis for lung cancer. 2-APB may potentially be used as new adjuvant drug candidate for co-treatment strategy with other chemotherapeutic agents that demonstrate similar characteristic as BZM in cancer therapy. The combination treatment as presented in this study provided insight into the development of a novel strategy for cancer with the potential translation into the clinics.

## Materials and methods

### Cell culture

H1975, A549, HeLa, U87, HepG2, MCF7, HCT8, BEAS-2B, LO2, and Lewis lung cancer cells (LLC-1) cell lines were purchased from American Type Culture Collection (Rockville, USA). These cell lines were authenticated by ATCC. Immortalized Atg7-wild-type and Atg7-deficient mouse embryonic fibroblasts (MEF) were kindly provided by Professor Masaaki Komatsu (Juntendo University, School of Medicine, Japan). All media were supplemented with 10% foetal bovine serum, 50 U/ml penicillin, and 50 μg/ml streptomycin (Invitrogen, UK). Cells were cultured at 37 °C in a 5% CO_2_-humidified incubator.

### Reagent and antibodies

The following reagents were used at doses indicated in the text and figures: BZM (Targetmol, T2399, USA), 2-APB (Meryer, M18515-1G, China), and HCQ (Santa Cruz, sc-215157, USA). The antibodies against LC3B (LC3-II) and Beclin-1 was purchased from Cell Signalling Technologies Inc (2775 and 3495, USA), and anti-β-actin was obtained from Santa Cruz (sc-47778, USA). ZyMaxTM TRITC-conjugated anti-mouse secondary antibodies (Invitrogen, PA1-28565, USA), and HRP-conjugated secondary antibodies were purchased from Cell Signalling Technologies Inc (2775, USA). Unless otherwise specified, all other reagents were purchased from Sigma-Aldrich (MO, USA).

### Immunoblot analysis

Western blot analysis was carried out following standard methods. Cells were lysed with RIPA lysis buffer (Cell Signalling Technologies Inc, 9806, USA). Protein detection was performed using Amersham Imager 600 (GE Healthcare Life Sciences) chemiluminescence (Invitrogen, USA). Band intensities were quantified by using the software ImageJ (NIH, USA). LC3 conversion was quantified by measuring band intensities of LC3-II and normalized to β-actin.

### MTT assay

Cell viability and the inhibitory concentration (IC) were determined by MTT assay. Cells were exposed to different concentrations of each compound or DMSO as a control for 72 h. After MTT incubation at 37 °C for 4 h, solubilization buffer (10% SDS in 0.01 mol/L HCl) was added for overnight incubation. Absorbance at *A*_570 nm_ was measured to evaluate the cellular enzymes reduction of tetrazolium salt into an insoluble formazan dye (cells viability). The percentage of viable cells was calculated using the following formula: Cell viability (%) = *A*_treated_ − *A*_background_/*A*_control_ − *A*_background_ × 100. Drug synergy was quantified according to the Chou–Talalay method for constant drug combination using the CompuSyn software^28^.

### Natural compounds screening procedure

800 compounds from Natural Products Collection (MicroSource Discovery Systems Inc., NP130501, USA) were screened in H1975 cell line by MTT. Firstly, the potential toxic compounds were discriminated (IC_50_ ≤ 10 µM). Then, half of the established dosage (½ IC_50_ value) was used in combination with BZM [0–100 nM]. The BZM sensitivity fold was calculated as IC_50 **BZM**_/IC_50 **COMBINATION**_.

### LIVE/DEAD cells analysis

Cell death was detected by LIVE/DEADTM Cell Imaging Kit (488/570) (Invitrogen, USA) according to the manufacturer’s instructions. Treated cells were stained with LIVE/DEADTM dye for 20 min. Cells were imaged by Olympus IX71 fluorescence microscope with FITC and TRITC filters consecutively. Green and red fluorescence images were merged and analyzed with cellSens Standard 1.8.1 software. The percentage of cell death was quantified by dividing the number of dying and dead cells (yellow and red fluorescence) by the total number of cells. A minimum of 1000 cells were scored from randomly selected fields.

### Endogenous LC3-II puncta quantification

In brief, cells on cover slips were treated, fixed with 4% paraformaldehyde (Sigma, 158127-3KG, USA) for 20 min, and then permeabilized with methanol for 2 min at RT. Samples were then incubated with anti-LC3 [1:200] in blocking buffer (5% BSA-TBST) overnight at 4 °C. After washing, cells were incubated with TRITC anti-mouse antibody [1:200] at 37 °C for 1 h in darkness. Finally, the coverslips were mounted onto microscope slides using FluorSave™ anti-fade mounting medium (Calbiochem, 345789, USA). Samples were imaged by widefield epifluorescence microscopy using Photometrics CoolSNAP HQ2 CCD camera on the Olympus IX71-Applied Precision DeltaVision restoration microscope (Applied Precision Inc, USA). All fluorescence images were deconvolved using DeltaVision algorithms (Applied Precision, Inc). The percentage of cells with endogenous LC3-II puncta was calculated following a specific autophagy guideline^55^ as the number of the cells with increased formation of punctate fluorescence dots (≥10 dots/cell) over the total number of cells in the same field. A minimum of 1000 cells from randomly selected fields were scored.

### Intracellular Ca^2+^ dynamic measurement

Intracellular cytosolic Ca^2+^ dynamic was measured using the FLIPR Calcium 6 Assay Kit (Molecular Devices, USA) according to the manufacturer’s instructions. FLIPR Calcium 6 stained cells were simultaneously treated on the FLIPR Tetra High-Throughput Cellular Screening System (Molecular Devices, USA). Real-time Ca^2+^ kinetic data were monitored for 30 min at 470/95 and 515/75 nm excitation and emission filters at 1-s reading interval. Fluorescent measurements were normalized to control (stained and non-treated cells).

### Mouse model

Eight-week-old C57BL/6 mice were purchased from The Chinese University of Hong Kong. All the experiments were carried out in accordance with the “Institutional Animal Care and User Committee guidelines” of the Macau University of Science and Technology. Mice were subcutaneously injected with Lewis lung cancer cells (LLC-1), randomly divided into five groups, and monitored for body weight and tumor volumes as previously described^56^. Survival rates were estimated using the Kaplan–Meier method. Five H&E-stained lung sections, taken at 50 μm intervals, were examined by microscope for metastatic lesions. Samples were imaged by Leica DFC310 FX camera and lung areas were calculated by Leica Application Suit V4.4 software. The percentage of metastatic lung area was calculated as metastatic burden area/lung area. Serum liver enzymes were measured using the Abbott Architect ci8200 analyser (Abbott Laboratories, USA).

## Electronic supplementary material

Chemical library screen

Synergic effect of 2-APB and BZM combination

Schematic representation of the C57BL/6 mouse model and the treatment regimen
