# Supplementary figures and images for: 2-Aminoethoxydiphenylborane sensitizes anti-tumor effect of bortezomib via suppression of calcium-mediated autophagy
Source: Cell Death Dis. 2018 Mar 2;9(3):361. doi: 10.1038/s41419-018-0397-0 (PMC5834458; doi:10.1038/s41419-018-0397-0)

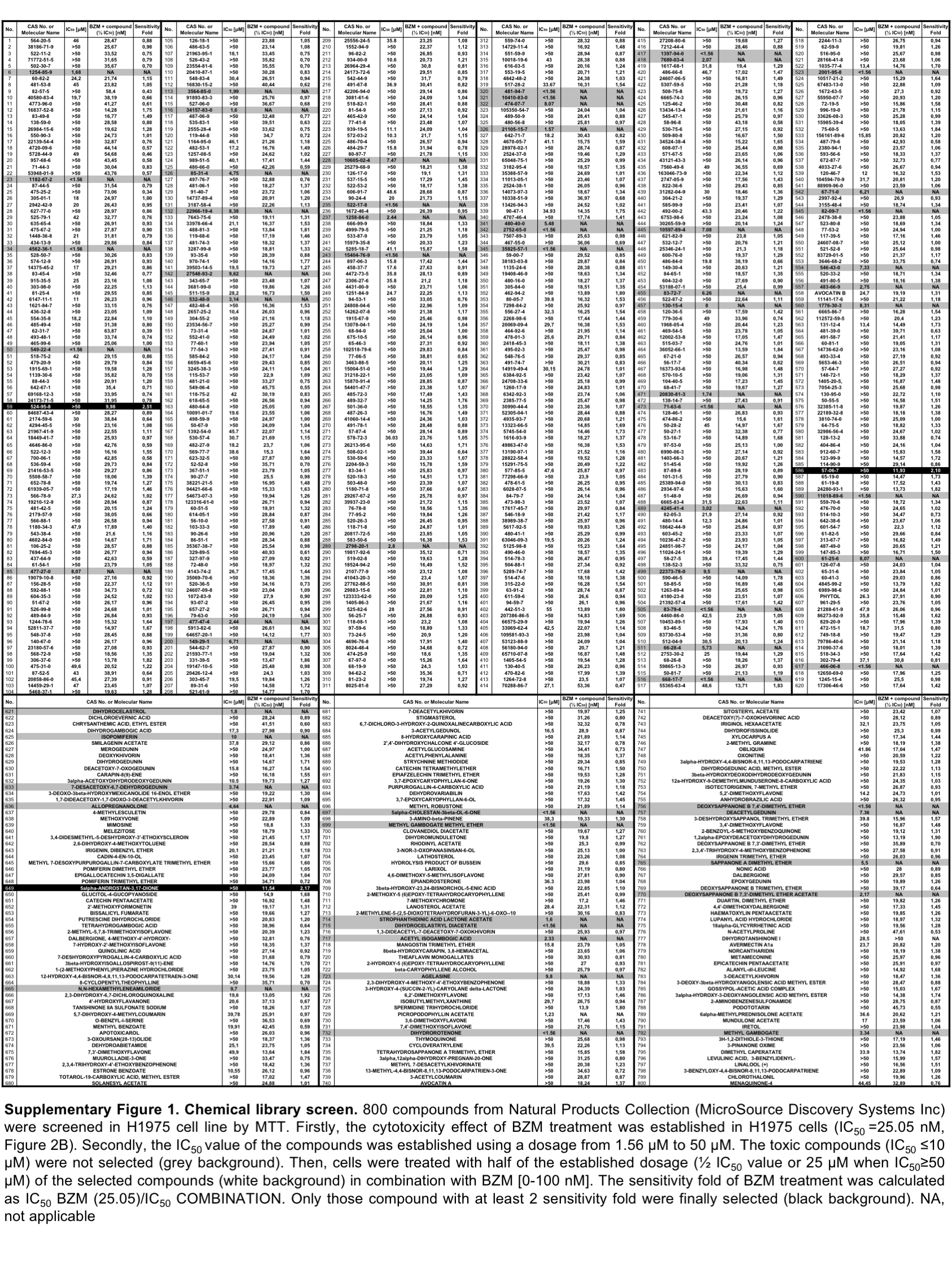

Supplement: Supplementary file 1 — Chemical library screen [file 41419_2018_397_MOESM1_ESM.png]

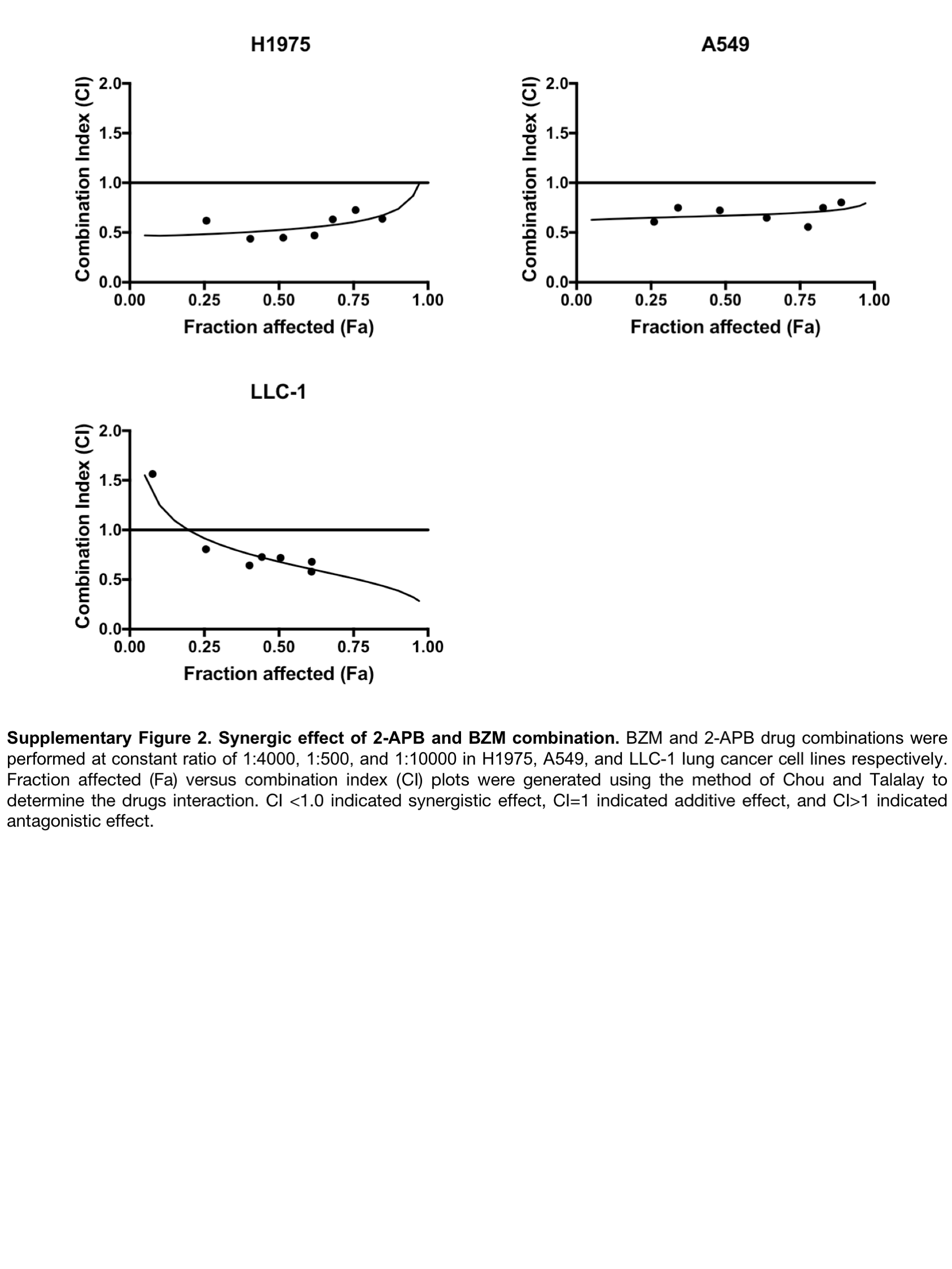

Supplement: Supplementary file 2 — Synergic effect of 2-APB and BZM combination [file 41419_2018_397_MOESM2_ESM.png]

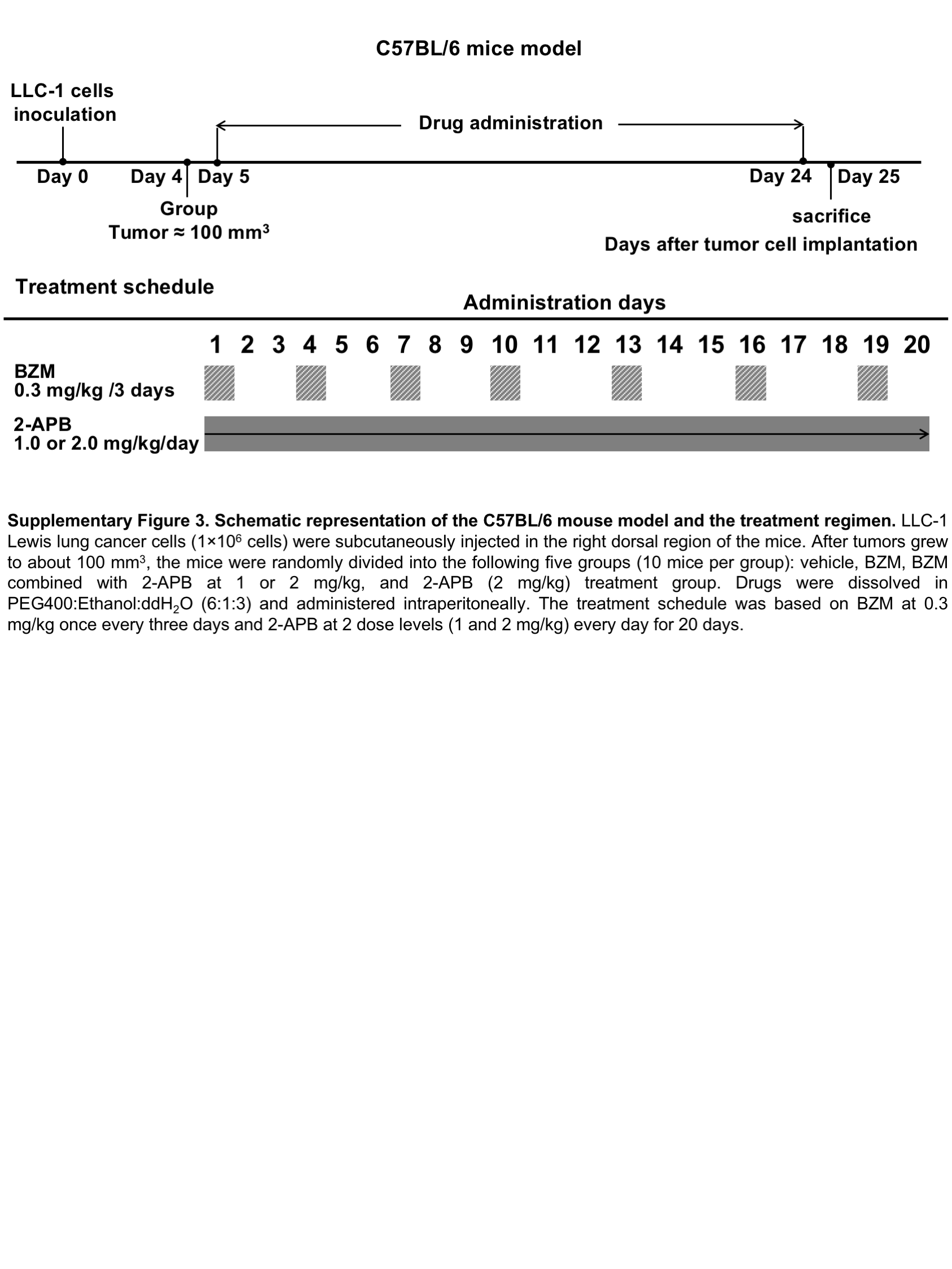

Supplement: Supplementary file 3 — Schematic representation of the C57BL/6 mouse model and the treatment regimen [file 41419_2018_397_MOESM3_ESM.png]
